# Real-time monitoring of calcium carbonate and cationic peptide deposition on carboxylate-SAM using a microfluidic SAW biosensor

**DOI:** 10.3762/bjnano.5.193

**Published:** 2014-10-22

**Authors:** Anna Pohl, Ingrid M Weiss

**Affiliations:** 1 INM – Leibniz Institute for New Materials, Campus D2 2, 66123 Saarbrücken, Germany; 2 Saarland University, Campus D2 2, 66123 Saarbrücken, Germany

**Keywords:** biomineralization, calcium carbonate, love-type surface acoustic wave, poly-cationic peptide

## Abstract

A microfluidic biosensor with surface acoustic wave technology was used in this study to monitor the interaction of calcium carbonate with standard carboxylate self-assembled monolayer sensor chips. Different fluids, with and without biomolecular components, were investigated. The pH-dependent surface interactions of two bio-inspired cationic peptides, AS8 and ES9, which are similar to an extracellular domain of the chitin synthase involved in mollusc shell formation, were also investigated in a biological buffer system. A range of experimental conditions are described that are suitable to study non-covalent molecular interactions in the presence of ionic substances, such as, mineral precursors below the solubility equilibrium. The peptide ES9, equal to the mollusc chitin synthase epitope, is less sensitive to changes in pH than its counterpart AS8 with a penta-lysine core, which lacks the flanking acidic residues. This study demonstrates the extraordinary potential of microfluidic surface acoustic wave biosensors to significantly expand our experimental capabilities for studying the principles underlying biomineralization in vitro.

## Introduction

Biomineralization is a natural process of global significance that involves the deposition of mineral ions under the control of biological organisms [1–7]. The interaction of proteins with minerals is one of the key regulatory elements in biomineralization processes, and many proteins involved in biomineralization exhibit molecular features that make them attractive models for materials science and nanotechnology [8–10]. Especially the protein fraction of mollusc shells is of interest for studying protein–mineral interactions because each mollusc shell contains a species-specific set of proteins which, in a collective manner, achieve superior materials properties in the final ceramic composite [11–13]. Molecular and phylogenetic investigations report that the differences between the assemblies of shell proteins from one species to another species can be tremendous [14–16]. This observation suggests that multiple interactions between the proteins are fine-tuned in relationship to the forming mineral phases [17–20].

Dissecting each single interaction event in a given biomineralization process poses some experimental challenges [21–24]. An investigation of biomolecular interactions, in such a process, requires quantifying the impact of different parameters under well-defined conditions [25–26]. Especially in the case of mineral precipitation, the required control over the process is limited when the reaction takes place closer to the solubility equilibria regime [27]. This could be one of the reasons why mechanistic insights into the functions of many biomineralization proteins are difficult to establish [9,28]. Mineral precipitates obtained in the presence of organic additives have been analyzed using an arsenal of characterization techniques including high-resolution X-ray and electron microscopy techniques [25]. However, many details about the dynamics of interfacial interactions – an intrinsic design feature of enzymatic catalysis common to all biological systems [4,26] – are still poorly understood as far as the design features of biomineralization proteins are concerned [14,19]. Especially in the field of biomineralization it is therefore of major importance to quantify biomolecular interactions as a solid–liquid system with high sensitivity.

Recent progress in the field of biosensors based on surface acoustic wave (SAW) technology has made it possible to perform experiments with very high sensitivity in extremely small volumes of liquid media [29]. Surface acoustic wave biosensors operate with different types of waves [30]. Not all of them are useful for fluidic applications due to an enormous energy loss [31]. The propagation of the surface acoustic wave is influenced by the adjacent medium. Phase and amplitude of the propagating wave vary as a function of viscosity changes related to mass deposited on the surface [32]. Advanced microfluidic biosensor technology is based on the propagation of a surface acoustic wave within a thin film [33]. Interdigital transducers on both sides of the sensor area give rise to an electrical field when alternating voltage is applied [34]. The electrical field is converted into mechanical stress that propagates through the material and generates the surface acoustic wave, which is converted at the opposite side of the sensor to an electrical signal by the direct piezoelectric effect. The ability to easily calibrate the system with high performance [35] is essential to ensure the observed changes in the acoustic wave are indicative of changes in the system free energy which changes as a function of mass transfer between the fluid and the sensor [33]. This opens the possibility to analyze the deposition of mass in real-time and as a function of organic additives under marker-free conditions [29].

The sensitivity of the new generation of microfluidic SAW sensors is about 4–5 times higher than that of quartz crystal microbalances with dissipation QCM-D [35]. Mass and viscosity changes can be continuously and simultaneously monitored in standardized systems, as long as the amplitude signal is strongly correlated with the viscosity of the fluid [36]. Recently, multichannel experiments became possible, enhancing the efficiency while expanding the experimental design options [37–38].

So far, the most common real-time assays with respect to the function of macromolecules involved in biomineralization are based on calcium titration in bulk environments [23,39]. Although these assays are perfectly suited to monitor the onset of early mineralization events including the formation of pre-nucleation clusters, the assay is restricted to comparably large volumes, which poses a problem for the study of biomineralization, where the amount of organic compounds is rather limited because of the low yield after purification, especially from native shell extracts [40], but even from recombinant sources [41–42].

The aim of the present study was to evaluate the suitability of microfluidic SAW biosensor systems with respect to elucidating the interaction between small biomolecules and calcium carbonate, one of the most common minerals in biomineralization processes. Acidic macromolecules comprise a significant fraction of the organic matrix of many mollusc shells [43–45], therefore a carboxylate-terminated self-assembled monolayer (COO-SAM) sensor chip was used to investigate under which conditions interactions related to organic biomolecules and calcium carbonate in the presence of carboxylate surfaces could be reproducibly quantified in real-time assays.

Here, we report a case study with calcium carbonate, both in pure aqueous systems and in the presence of citric acid. We also investigated the two cationic peptides ES9 (sequence, N→C: EEKKKKKES) and AS8 (sequence, N→C: AKKKKKAS) [46]. The ES9 peptide is derived from E22, one of four major extracellular loops of an enzyme involved in biomineralization [20,47]. Previous experiments suggest that self-assembly of E22 is fine-tuned in accordance with pH changes that may occur as a function of the mineral precipitation and dissolution [48]. Since no equivalent real-time information regarding the deposition of calcium carbonate on carboxylate-SAMs below the equilibrium solubility is available from the literature, we started our investigation and described our results in detail with the particular aim to inspire similar investigations in biomineralization research in the future. A standard database of the function of organic molecules, peptides and proteins in mineralizing systems will be a major achievement of global significance for materials science, biomedical engineering and bioinspired nanotechnology.

## Results and Discussion

Using a standard microfluidic surface acoustic wave (SAW) biosensor system equipped with commercially available COO^−^/H_3_O^+^-SAM (COOH-SAM, in the following is termed COO-SAM) sensor chips, we monitored the phase and amplitude signals as a function of time. The influence of the concentration of calcium carbonate relative to the solubility equilibrium and the flow rate on the SAW biosensor phase and amplitude signals was investigated in real-time. The influence of organic molecules is studied in two different systems: First, citric acid in aqueous solution was investigated in a flow channel pre-incubated with calcium carbonate. Second, two peptides (pI 10 and pI 9) in glycylglycine buffer (Gly–Gly) were investigated at three different pH values between pH 7.75 and pH 9.0 in order to learn about their interaction with charged surfaces. The aim of this study was to evaluate the competitive non-covalent interactions of peptides and buffer substances at the liquid–solid interface. For standardization, commercially available COO-SAMs were used to mimic negatively charged surfaces, which similarly occur in natural biomineralization processes. The sequence of injections into the respective flow channels is an essential part of each experiment and the tables are presented in Supporting Information File 1.

### Calcium carbonate in water

**Figure 1:**
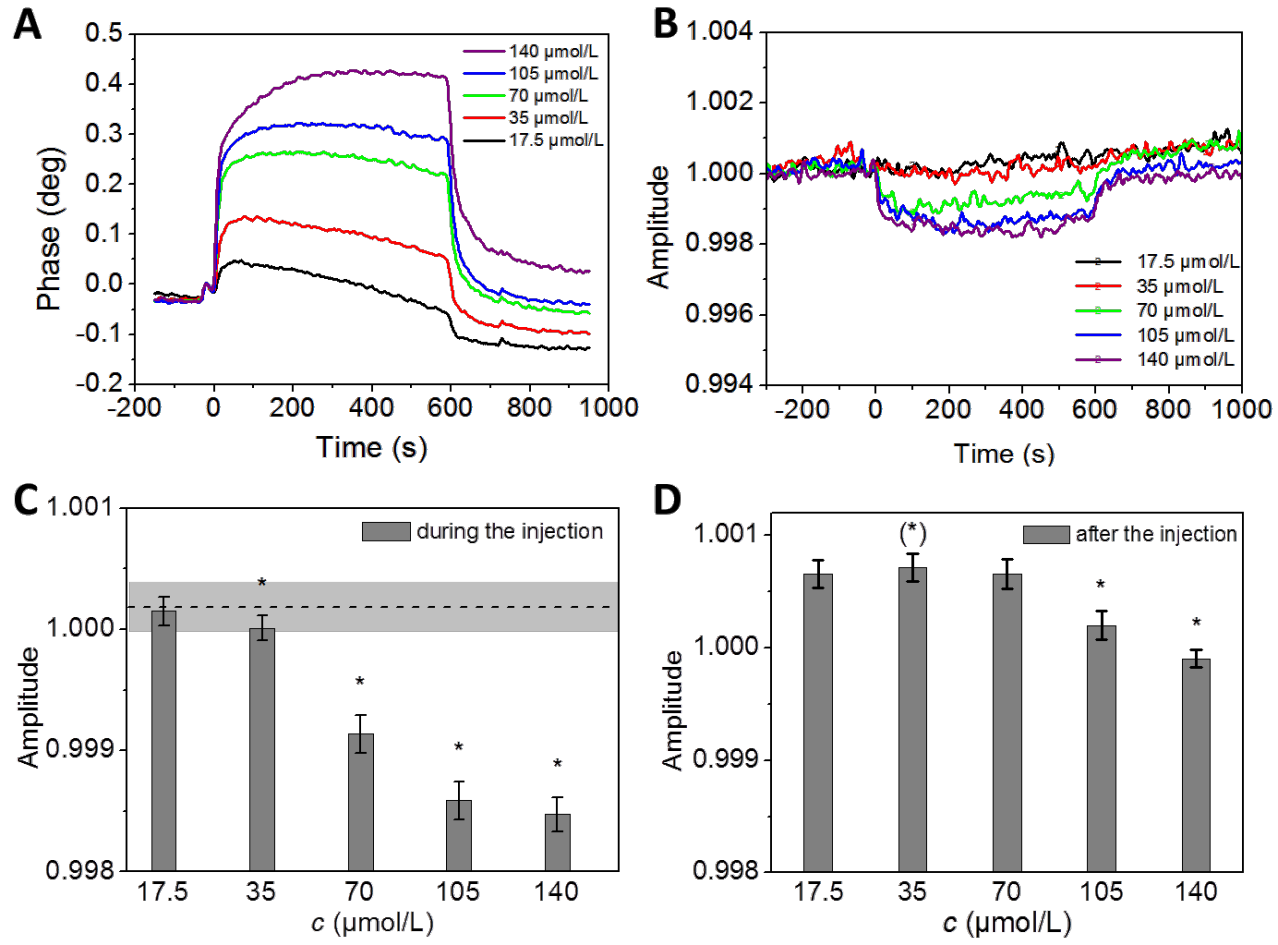
Phase and amplitude signal of microfluidic experiments on COO-SAM with calcium carbonate in pure water at different concentrations. Calcium carbonate solutions of 140 µmol/L (equilibrium saturation level, violet), 105 µmol/L (blue), 70 µmol/L (green), 35 µmol/L (red), 17.5 µmol/L (black) were injected in a sam^®^X system and interactions with a COO-SAM sensor chip were monitored in real-time. The phase vs -time plot visualizes the change of the phase signals (A) and the corresponding amplitude signals (B) of the sensor during the injection of the calcium carbonate solutions (0–600 s) and pure water (600–1000 s). (C), Statistical evaluation of averaged amplitudes prior to (shaded box) and during injections as shown in (B). (D), Comparison between the amplitude signals after the injections as shown in (B). There is a significant difference between the lowest concentration (*c* = 17.5 µmol/L) and *c* = 105 µmol/L or, *c* = 140 µmol/L, indicating that long-lasting residence of calcium carbonate at the carboxylate-SAM interface occurs at *c* > 70 µmol/L but far below the calculated equilibrium in solution. Note that the amplitude signal during the injections (C) remained unchanged only for the lowest concentration (*c* = 17.5 µmol/L).

The first series of experiments are designed to test whether dynamic interactions between calcium and (bi)carbonate ions (hydrogen carbonate and/or carbonate) with a COO-SAM surface can be monitored in real-time and whether or not the interaction depends on the concentration, which was adjusted to fractions of the solubility equilibrium concentration. Therefore, 140 µmol/L calcium carbonate in pure water was diluted to 105 µmol/L, 70 µmol/L, 35 µmol/L, 17.5 µmol/L. Deionized water was used as the running buffer in these experiments and calcium carbonate solutions were sequentially injected, all into the same channels of a four-channel sensor chip, starting with the lowest concentration (Supporting Information 1, Table S1). In between the injections, deionized water (the "running buffer" in this case) was supplied to all channels of the sensor chip for five minutes. Figure 1 shows the phase and amplitude signals of the SAW sensor in response to the calcium carbonate injections at different concentrations.

The phase signal as a function of time (Figure 1A) was nearly the same for all concentrations tested in this experiment. At time = 0 seconds the injection started, lasting for 600 seconds in this case. After an initial fast increase, the signal remained more or less constant at maximum values, dependant on the different calcium carbonate concentrations. The 140 µmol/L solution reaches 0.44°, whereas the 17.5 µmol/L solution achieves the lowest maximum phase value with only 0.05°. The baseline noise, according to the manufacturer of the instrument, is <0.05° phase (RMS), the baseline drift typically <0.01° phase/min. The signal decreased when the channel is flushed with an injection of water and reached a nearly constant value, which differed from the starting conditions up to about 0.04°, corresponding to about 10% of the maximum phase value reached during the continuous calcium carbonate flow. It is unclear, how stable the interaction with the COO-SAM during the calcium carbonate injection would be. The obtained value could just indicate a more or less stable equilibrium between precipitation and dissolution from the surface. The final value, which is reached after pure water is injected, very likely represents the complete dissolution of a precipitate temporarily attached to the COO-SAM. If this is indeed true, it would represent a calcium carbonate precipitate reversibly formed under conditions far below the solubility equilibrium, thus providing an opportunity to study the function of biomineralization proteins under conditions where spontaneous mineral nucleation should not occur. This would, in turn, represent a new degree of freedom in biomineralization research.

The response of the amplitude signal to the injection of calcium carbonate solutions is in the range of 0.998–1.001 a.u. (compare also with citrate experiments described in the following section). The amplitude data presented in Figure 1B are evaluated with respect to significant differences before and after the injection of calcium carbonate (Figure 1C). The amplitude signals decrease with increasing calcium carbonate concentrations of at least 35 µmol/L. The data sets of the different calcium carbonate concentrations were compared after the injections were followed by a constant flow of deionized water (Figure 1D). After equilibration, amplitude signals of 105 µmol/L and 140 µmol/L remain low, whereas the 70 µmol/L reaches the same range as the 17.5 µmol/L (Figure 1D). The relatively high amplitude signal corresponding to 35 µmol/L indicates additional drift effects. In summary, it can be concluded that the temporary interaction with the sensor chip is concentration dependent. A minimum concentration of 35 µmol/L is required. After the injection of deionized water (Figure 1A, 600–1000 s), the 105 µmol/L or 140 µmol/L calcium carbonate injections apparently lead to stable calcium deposits on top of the COO-SAM. The lower concentrations are similar to 17.5 µmol/L calcium carbonate, which appears inert during the injection. There is no long-term effect. This observation raises the question of whether there is a structural difference between calcium deposits on COO-SAMs obtained at higher versus lower concentration levels. Such structural differences could account for the sustained stability of the layer as indicated by both the phase and the amplitude signals.

Since even the smallest change in concentration causes a discernable change in the phase signal (Figure 1A), this biosensor system is suitable to detect biomolecular interactions with calcium carbonate in the range of *n*(CaCO_3_) ~10^−12^ mol to 10^−11^ mol. One microfluidic channel has a flow cell height of 2 × 10^5^ nm and a volume of 1.2 µL. The sensitive area of the chip is 5 × 10^6^ nm × 1.2 × 10^6^ nm and the cross-sectional area of the flow channel is 0.24 mm^2^. This would correspond to ~2 × 10^13^ to 3 × 10^13^ elementary cells of calcite, assuming that these would form a homogeneous monolayer on the sensitive area of one sensor channel. The commercial SAM's used here were not specifically characterized with respect to the molecular ordering. It would have exceeded the scope of the present study to investigate the growth kinetics of differently oriented calcium carbonate crystals [44–45], and to evaluate the homogeneity of the calcium carbonate films deposited on the sensor chip.

The injection of pure water did not result in a complete reduction of the phase signal. This suggests that calcium ions and/or calcium carbonate are not completely removed from the sensor chip surface. It seems that part of the previously formed layer is stable with respect to the water interface at a given flow rate, depending on how closely the concentration of the dissolved calcium carbonate matches the solubility equilibrium during deposition. The relatively small changes in the amplitude signals during the injection indicate that the overall viscosity effect on a COO-SAM is small in the case of calcium and (bi)carbonate ions in aqueous solution.

### Flow rate dependence

**Figure 2:**
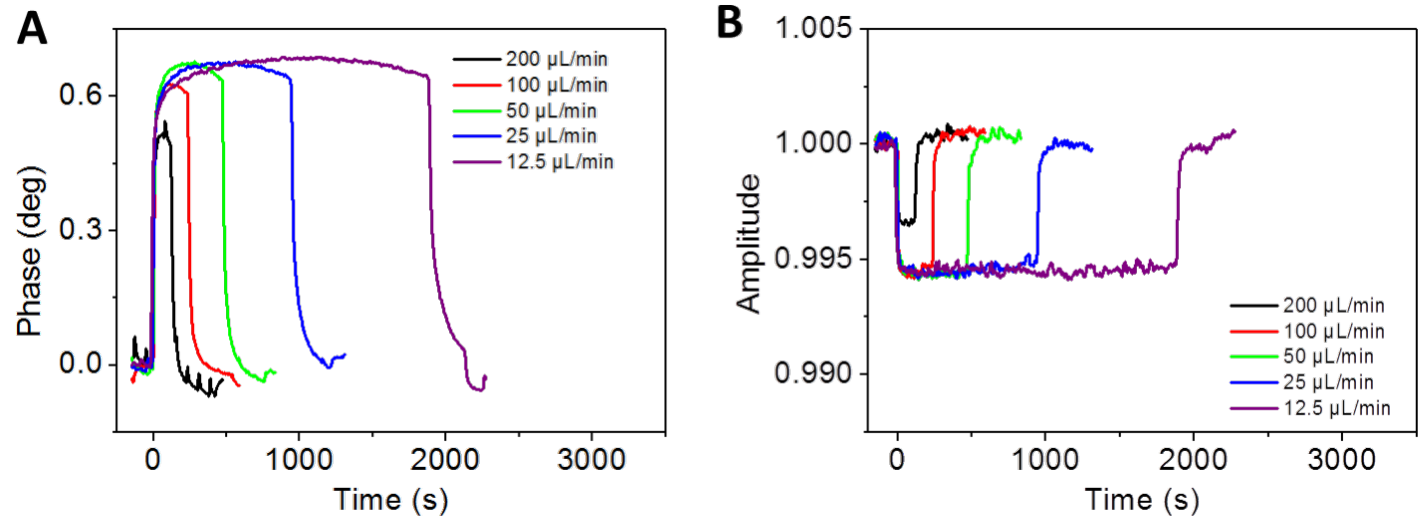
Flow-rate dependency of calcium carbonate interactions with COO-SAM chip. Phase vs time plot (A) and corresponding amplitude vs time plot (B) for different flow rates. A constant volume of 400 µL calcium carbonate in pure water (140 µmol/L) was injected, followed by pure water for 5 minutes.

The sam^®^X microfluidic system was designed for flow rates in the range between 12.5 µL/min and 300 µL/min. The influence of different flow rates on the interaction between ions derived from calcium carbonate in aqueous solution and the sensor chip surface was tested at flow rates of 200 µL/min, 100 µL/min, 50 µL/min, 25 µL/min and 12.5 µL/min. At each time, 400 µL of the calcium carbonate solution was injected. The system was allowed to equilibrate for 5 minutes between individual experiments with pure water at the respective flow rates (Supporting Information 1, Table S2). Figure 2 shows the phase vs time and amplitude vs time plots obtained when a calcium carbonate solution at solubility equilibrium was injected at different flow rates.

The initial slope and overall shape of the phase signal vs time is independent of the flow rate. A maximum phase value of ~0.7° is reached at flow rates up to 50 µL/min. When the experiments are performed at flow rates 100 µL/min and 200 µL/min, the maximum phase values are in the range 0.5–0.6°, which indicates that the threshold for flow rate independence is between 50 µL/min and 100 µL/min. The little irregular peaks in the phase signal obtained for 200 µL/min flow rates can be attributed to the pumping procedure which is a common phenomenon when the flow rate is high. The volume of the pump is 250 µL, and each time when this volume is refilled it causes artifacts in the signal as observed here.

Since the flow rate determines the duration of the injection, we chose to keep the injection volume and thus the total amount of injected calcium carbonate constant. If the phase signal at flow rates up to 50 µL/min, as shown in Figure 2A, were displayed at stretched time intervals, they would correspond to each other in terms of maximum height and curvature. There is no flow rate dependency as long as flow rates are kept below 50 µL/min. The highest flow rate tested (200 µL/min) yields the lowest phase signal at approximately 0.5°. Flow rates below 50 µL/min reach the maximum phase at values between 0.6 and 0.7°. In accordance with these observations, all subsequent experiments are performed at flow rates equal to or less than 50 µL/min. The maximum phase values are reached after approximately 200 µL injection volume. This observation suggests that the time course of the interaction depends also on the total amount of calcium and/or (bi)carbonate ions introduced to the sensor surface. It is interesting to note that calcium carbonate surface interactions seem to be less well controlled at flow rates higher than 50 µL/min. One can assume that, at higher flow rates, the interaction of calcium and/or (bi)carbonate does not reach the maximum capacity of the sensor surface.

### Calcium carbonate and citric acid interactions

In order to explore the possibility of calcium carbonate removal from the sensor chips as seen in the previous experiments, the COO-SAM surfaces of the sensor chip were treated with different volumes of 1 mM citric acid. A solution of calcium carbonate at solubility equilibrium (140 µmol/L) was used as the running buffer and different amounts of citric acid (100 µL, 200 µL, 400 µL) were injected successively. The flow rate was kept constant at 50 µL/min. Pure water was also injected for comparison. Table S3 (Supporting Information File 1) shows the sequence of injections and experimental details.

**Figure 3:**
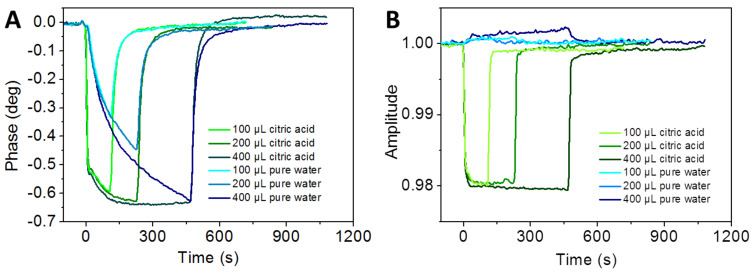
Monitoring of citric acid on calcium carbonate treated sensor surfaces. Phase (A) and corresponding amplitude (B) signal versus time for different injection volumes of 1 mM citric acid. Citric acid and pure water differ in their interaction with sensor chip surfaces in the presence of calcium carbonate.

The time course of the phase signal for the duration of each injection volume was similar for all injected volumes of 1 mM citric acid (Figure 3A).

The phase signals of pure water and citric acid experiments are displayed in an overlay, adjusted to phase = 0° and time of injection = 0 seconds. The injected volume determined the length of each injection. There is almost no change in the phase signal of the three injections of pure water (Figure 3A), all of them have the same negative gradient during the injection. The phase signal decreases with time in an almost parabolic shape. As soon as the injection ends there is a fast increase in the phase signal until it reaches the original value. Phase signals at the end of the injection period reach a certain minimum value, depending on the volume and hence the duration of the injection. Nevertheless, all phase signals of the citric acid injections decrease very rapidly with nearly the same negative slope, which decreases with time until a constant minimum value is reached (Figure 3A, 200 µL and 400 µL citric acid). As soon as the injection is finished, the phase signal increases very rapidly until the original value close to 0° is reached. In the case of pure water, an injection volume of 400 µL is required to reach the minimum phase value of the citric acid experiment.

Figure 3B shows the corresponding amplitude vs time plot. Almost no amplitude changes occur when different volumes of pure water are injected. In contrast, the amplitude signals for the injections of citric acid decrease immediately after injection and remain at a constant minimum value as long as citric acid is injected. When the injections stop, the amplitude signals return quickly to the original value of 1.00 (a.u.). The volume of citric acid and thus the total amount of organic molecules has little effect in this case. The volume of the injected solution changes only the duration of the injection period and does not change anything in the behavior of the amplitude signal. Experiments with 10 mM citric acid show that the phase signal behave more or less the same, but the minimum phase value attained during the injection is much lower than the phase shifts caused by 1 mM citric acid solution.

**Figure 4:**
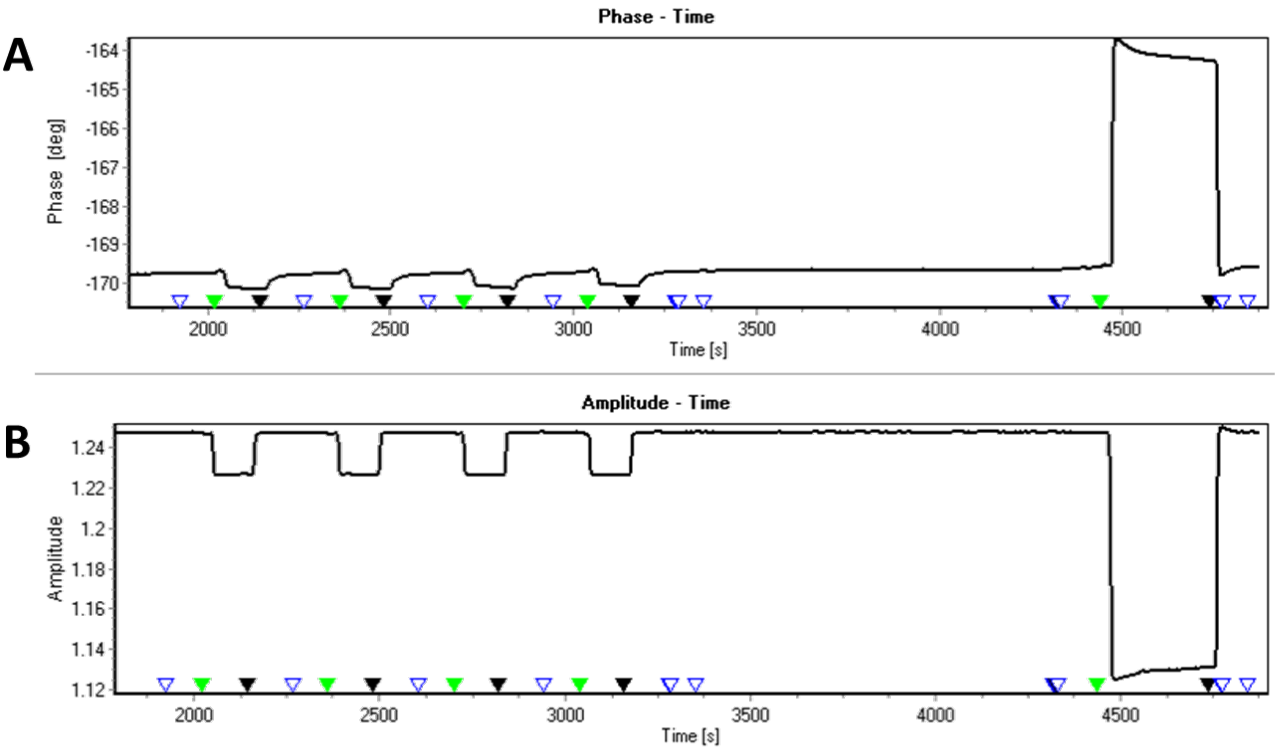
Experimental determination of the mass normalized phase signal in a citric acid/calcium carbonate system. A calcium carbonate running buffer was used here. Citric acid was injected for 120 seconds, followed by regeneration intervals of several minutes. Finally, 5% glycerol were injected. Green triangles, injection start; black triangle, end of injection.

The decrease in the phase signals observed for citric acid and pure water corresponds to a loss of mass on the surface of the sensor chip. Based on the time-course of the phase signals we assume that calcium carbonate originating from the running buffer is removed from the sensor surface by pure water and, even faster, by citric acid. Furthermore the time-course of the removal of calcium carbonate from the surface depends on the total volume of the citric acid injected. A certain amount of citric acid is required to reach the equilibrium level that leads to a depletion of mass, represented by the phase signal. A maximum turn-over is reached at 200 µL of citric acid or 400 µL of pure water. The observation that the changes of the phase signals caused by citric acid occur much faster than the changes of those induced by pure water injections indicates the removal by citric acid is more efficient. This is in perfect agreement with expectations, confirming that an online monitoring of the sequence of events in the removal of calcium and/or (bi)carbonate ions from COO-SAM surfaces by citric acid in comparison to water is possible. However, as shown in Figure 3B, the viscosity effect was not observed to follow the trend of the observed mass loss for the two different solvents (phase signal, Figure 3A). Citric acid and pure water induce a mass loss from the calcium carbonate treated COO-SAM sensor chip, but only citric acid shows a significant viscosity effect. Whether the temporary residence of citric acid molecules on the calcium carbonate layer can explain this behavior remains subject to further investigations. When 10 mM citric acid was used, the viscosity effect was even more pronounced and prevented reliable phase measurements (data not shown). We also tested the applicability of mass normalization based on integrated features provided by the commercial biosensor sam^®^X and the software SenseMaster [49]. An example for the experimental procedure is described in Supporting Information File 2. The phase vs time and amplitude vs time plots are shown in Figure 4.

**Figure 5:**
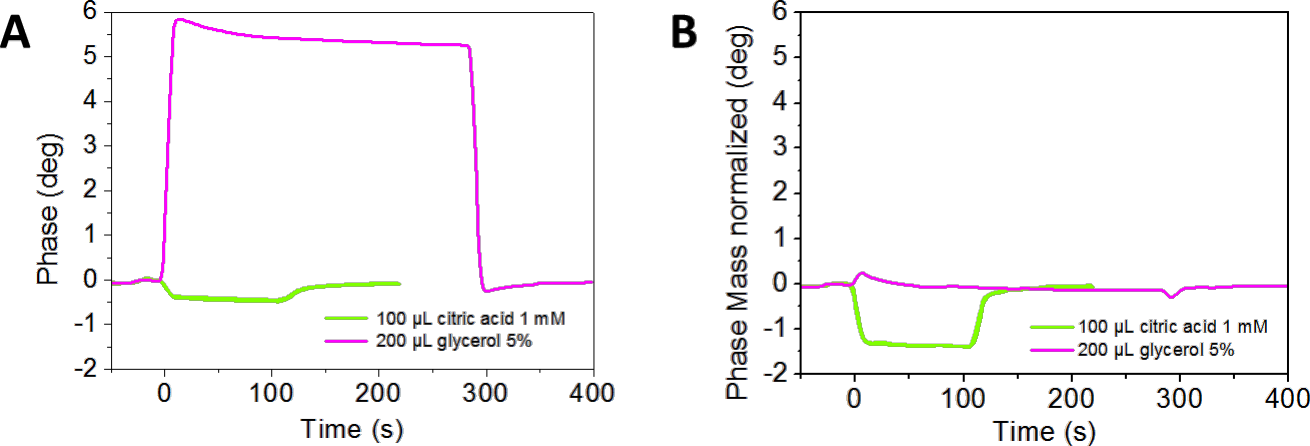
Comparison between the original phase signal and calculated mass normalized phase signal. The time-courses of all signals are aligned according to the injection start. The green lines represent an overlay of 4 citric acid injections as indicated in Figure 4. Note that these signals are extremely reproducible. The line in magenta represents the glycerol injection. (A), original phase signal; (B), calculated mass normalized phase signal. The glycerol signal in (A) was used for the software-automated calculation of all signals shown in (B).

Experimentally, the procedure is very fast and easy to perform, since the experiment is simply followed by an injection of an appropriate viscous substance. In this case, 5% aqueous glycerol was used. The calculation of the normalized mass signal is shown in Figure 5.

First, the "SenseMaster" software calculates a correction factor based on the phase difference between two values prior to and after the injection of the reference substance, arbitrarily selected by the user. For example, in the case of *t*_1_ = 4200 s, and *t*_2_ = 4700 s (Figure 4A), the phase difference for glycerol is 5.4° (Figure 5A). In the second step, this factor is used for calculating the normalized phase where, in principle, the "viscosity effect" should be eliminated. In the case of glycerol, the phase is normalized to −0.075° ± 0.074° (Δ*t* = −100–400 s, Figure 5B). The calculated phase ("mass normalized", Figure 5B) for calcium carbonate is decreased by the injection of citrate from −0.439° ± 0.015° to −1.363° ± 0.015° (mean values of 4 individual injections between 30–100 seconds). The respective amplitude signals are shown in Supporting Information File 2. These observations would be consistent with a relative mass loss, which is induced by the injection of citrate. This experiment provides first evidence that calcium carbonate interacts with the COO-SAM surface in the range of the solubility equilibrium, and that the molecular interactions could be quantified to some extent.

The procedure was experimentally established for biomolecular systems in order to evaluate the underlying theoretical calculations [49]. The same procedure, however, must not be used for calculating viscosity and mass effects in mineral systems, unless further experiments provide the evidence that interference with density and/or viscosity effects in such complex systems are negligible. Unless specifically mentioned, all data sets shown in this paper represent the original measurements for both, phase and amplitude obtained without viscosity correction procedures.

### Cationic peptide interactions on COO-SAM

Two positively charged peptides, AS8 and ES9, were previously shown to produce calcium carbonate precipitates with characteristic complex morphologies as a function of pH [46]. We are interested to see whether these peptides also interact with negatively charged surfaces such as COO-SAM as a function of pH. We are also interested to see whether the molecular features of these peptides (pI 9, pI 10) could be differentiated by using the microfluidic biosensor system sam^®^X. The buffer substance glycylglycine (Gly–Gly) is used to maintain the pH values 7.75, 8.2 and 9.0, similar to the previous studies [46,48]. The peptide AS8 represents a modified version of the peptide ES9 with only two differences in the length and in the substitution of two glutamic acid residues by alanines [46]. Different concentrations of peptides (0 µM, 50 µM, 100 µM, 200 µM) are dissolved in Gly–Gly buffers and adjusted to pH values 7.75, 8.2 and 9.0. The highest concentration (200 µM) was used for the experiments presented here. In Table S4 (Supporting Information File 1), a representative experiment with peptides ES9 and AS8 in Gly–Gly buffer is listed. Experiments at pH 7.75, pH 8.2 and pH 9.0 were performed analogous on the same biosensor chip using separate channels. The flow rate was 40 µL/min and the running buffer was the respective Gly–Gly buffer. Between the injections of the peptides EDTA was injected. Volumes of 200 µL were injected if not otherwise indicated.

**Figure 6:**
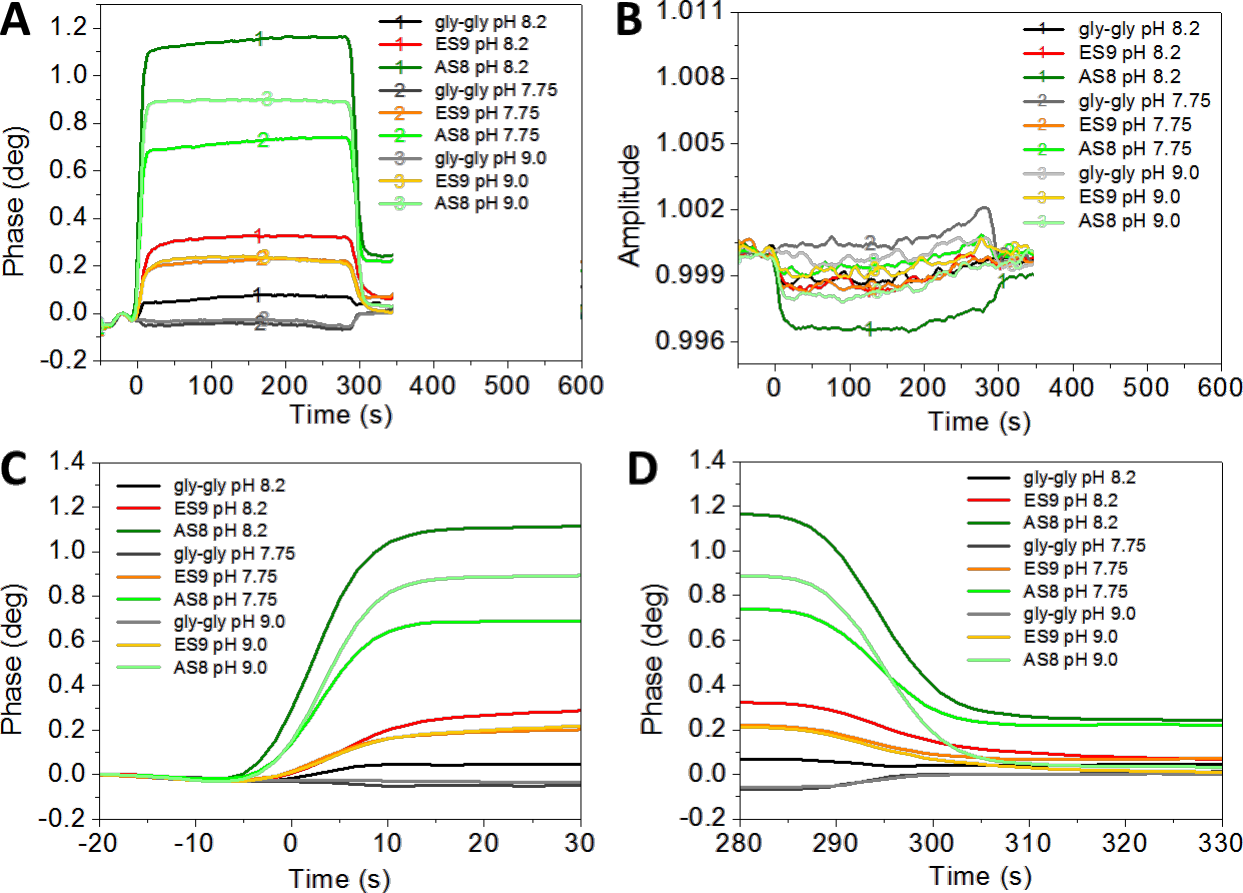
Monitoring of cationic peptides ES9 and AS8 on COO-SAM sensor surfaces. Phase (A) and corresponding amplitude (B) signal vs time for Gly–Gly pH 7.75, pH 8.2, or pH 9.0 with 200 µM peptides ES9 and AS8 (injection: 0–300 s). Graphs were produced by an overlay of data-sets (rows 2, 4, 6 of Table S4, Supporting Information File 1) at the time of injection (setpoint: 0 s). For clarity, the phase signals at the onset (C) and offset (D) of peptide injections are shown in time intervals of 50 seconds.

The three experiments with the different pH values are performed sequentially on separate channels of the same sam^®^X sensor chip. It was necessary to adjust the entire system to the same buffer conditions for Gly–Gly buffers pH 7.75, pH 8.2 or pH 9.0, in order to avoid uncontrolled response of the SAW sensor system when switching from the running buffer to injection mode. The peptide solutions were sequentially injected on the same channel, first ES9 and then AS8. The phase signals for 200 µM peptide injections according to Table S4 (Supporting Information File 1) are shown in Figure 6.

**Figure 7:**
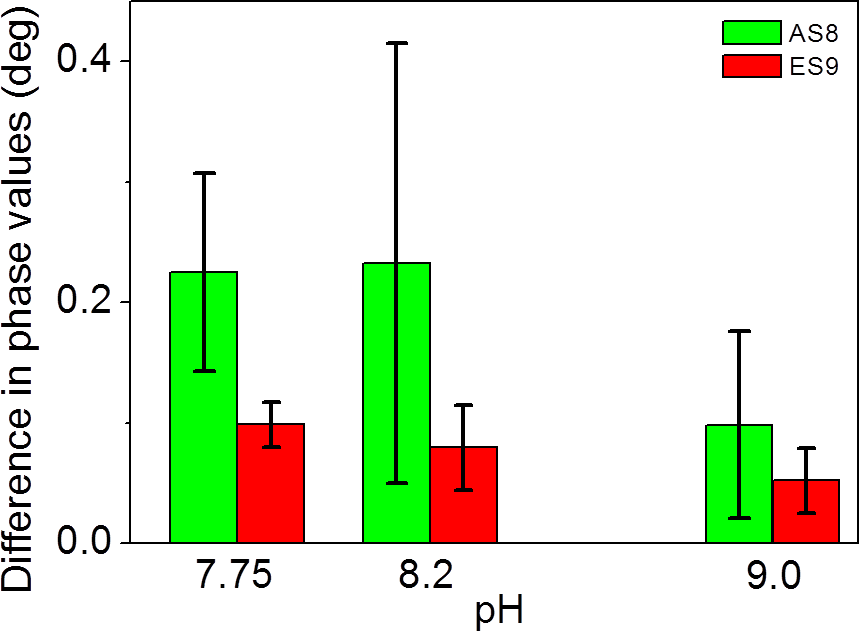
Evaluation of the phase difference prior and after injection of peptides as shown in Figure 6 A,C,D. For experimental values, see Supporting Information File 3.

The observed maximum phase shift values depend on the pH. For both peptides, the maximum phase shifts are observed for pH 8.2. Taking all experiments into account, it is 1.4° ± 0.3° in the case of AS8 and 0.6° ± 0.5° in the case of ES9. The latter only reaches 0.24° ± 0,03 at pH 7.75 and 0.25° ± 0.03° at pH 9.0. Peptide AS8 reaches maximum values of approximately 0.78° ± 0.03° at pH 7.75 and 0.96° ± 0.09° at pH 9.0. These observations suggest that pH 8.2 provides conditions for both, the cationic peptides and the carboxylate-terminated COO-SAM, to have the maximum surface interaction. However, the maximum value represents steady-state conditions as long as peptide is continuously supplied. We also evaluated the difference between the initial phase value (setpoint: 0°) and the phase values at the time = 310 seconds, 10 seconds after the injections finished (Figure 6D). This difference indicates the amount of peptide that remains stably adsorbed to the surface. As shown in Figure 7, there is a trend for peptide ES9 indicating the peptide interaction is decreasing with increasing pH.

At pH 9, the interaction is lowest for both peptides. This is in agreement with simple calculations according to the Henderson–Hasselbalch equation [48], since most of the lysine residues are neutral under these conditions. The presence of negatively charged glutamic acid residues in peptide ES9 leads to a reduction in the interaction with carboxyl-terminated SAM. As Figure 7 also shows, the AS8 has a larger variation in the phase difference, especially at pH 8.2. Previous results suggested that the peptide AS8 is not as well suited to gain control over calcium carbonate precipitation and crystal growth as the peptide ES9 [46]. Further work is required to elucidate the exact mechanisms in the case of AS8/CaCO_3_ or ES9/CaCO_3_ composites. However, the results as presented here suggest that the previously predicted pH-dependent interfacial interactions of each peptide species, which is intimately related to its sequence and pI, can be quantified by comparative sam^®^X biosensor experiments in order to predict which peptide sequences are more suitable to gain control over biomineralization in vitro and in vivo.

**Figure 8:**
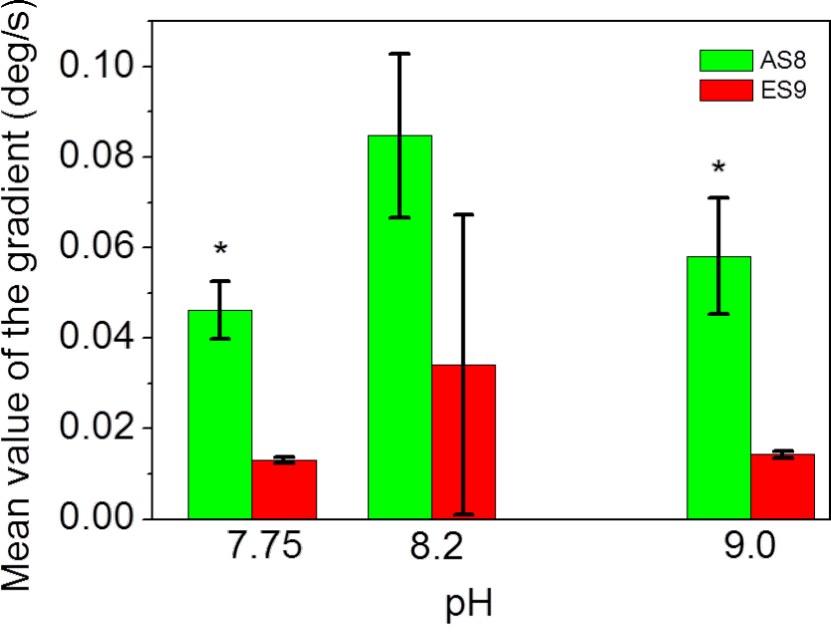
Evaluation of the slope observed in peptide experiments as shown in Figure 6C. For experimental values, see Supporting Information File 4.

The initial slope prior to reaching the steady-state level (Figure 6A,C) is steeper for peptide AS8 (calculated pI ~10.4) as compared to the peptide ES9 (calculated pI ~9.2). We evaluated both, the slopes at the time of injection (Figure 6C), and after the injection is finished (Figure 6D). The results for the injection of the peptides are shown in Figure 8.

This graph demonstrates that the two peptides, which differ in pI due to the exchange of glutamic acid residues (glu, E) against alanine residues (ala, A), flanking the penta-lysine core (N-EEKKKKKES-C vs N-AKKKKKAS-C) interact with carboxylates on the SAM-biosensor chip surface in a different way. The presence of the glutamic acid residues stabilizes the mode of interaction with the charged surface. When lysine residues are flanked by neutral amino acids, the interaction seems more likely to depend on pH. In the case of ES9, which represents the evolutionary optimized conserved sequence derived from the natural biomineralization system, the interaction seems to be less dependent on pH, suggesting a more robust control mechanism for the interfacial interaction of the tissue with the forming biomineral.

It remains subject to speculations whether, for example, pH-dependent peptide folding could favor interpeptide or intrapeptide interactions, which could also reduce the potential for interaction with the negatively charged COO-SAM surface. It was clearly shown in this experiment that the Gly–Gly buffer does not interfere significantly with the interfacial interactions of the positively charged penta-K (penta-lysine) peptides, and can therefore be regarded as a neutral agent which mainly serves for maintaining the pH. The fact that phase signals differed relatively to each other as function of pH further demonstrates that the buffer capacity of the chosen Gly–Gly system is sufficient to probe various environmental conditions in a convenient way.

## Conclusion

Biomineralization processes are very complex systems. They depend on evolutionary conserved, collective biomolecular interactions in fluidic systems, which are even more difficult to mimic when minerals are deposited or dissolved. Recently developed microfluidic biosensor systems provide promising perspectives for quantifying such biomolecular interactions under defined conditions. The biosensor sam^®^X (SAW Instruments, Bonn, Germany) used in this study combines a four channel microfluidic system with SAW detection and is equipped with an efficient software-driven access for automated sample processing and data analysis. The injection of a glycerol reference is a useful technique to separate the mass signal from the viscosity effect of the surrounding medium. In most cases the latter is represented by the amplitude signal. The mass deposited at the surface can be calculated from the viscosity normalized phase signal. As demonstrated here, this effect was marginal in the case of the purely aqueous calcium carbonate system. However, in the case of organic additives this may as well change. As a case study for a more complex system in biological buffers at various pH, we chose a peptide which was taken from an extracellular domain of the chitin synthases involved in biomineralization. A control peptide was designed with a slightly higher pI from glutamic acid residues, flanking a penta-lysine motif, were replaced by alanines. The pH-dependency of these peptides' interactions with calcium carbonate precipitation was previously demonstrated [46]. The in vitro systems tested so far were operated under saturated conditions by means of sodium bicarbonate and calcium chloride, thus suffering from additional salt effects. Only the finally obtained precipitate was analyzed further. The microfluidic biosensor experiments, in contrast to the previous study [46], bear the advantage of detecting mineral interactions prior to reaching the solubility equilibrium, as demonstrated here for the first time. So far, there are not many studies on biomineralization performed using microfluidic SAW sensor techniques [25]. We therefore started with calcium carbonate deposition on negatively charged standard SAMs. Our present results confirm that there are many parameters that determine the interaction of dissolved calcium carbonate with the carboxylate surface. The investigation of the cationic peptides AS8 and ES9 was therefore limited to the evaluation of the pH-dependency of COO-SAM interaction, but still in the absence of calcium carbonate. This system provides the chance to fine-tune parameters such as temperature, which also influences the solubility equilibrium, and flow rates. The influence of the buffer system can be monitored along with the samples of interest for direct comparison on the same chip. On the other hand, further experiments are necessary to interpret, for example, the difference in the dissolution of calcium carbonate by citric acid versus pure water. One has to be careful in terms of interpreting the large negative amplitude signal of the citric acid experiments mainly as a viscosity effect as suggested by the manufacturer (SAW Instruments). The normalized mass signals presented in this study certainly require additional work to establish a reliable sensitivity factor (Δ*S*, [µg^−1^·cm^2^·°]). As demonstrated here, the calcium carbonate concentration plays a significant role in the interaction with the carboxylate SAM, whereas the flow rate does not really matter unless it is very high. The possibility to investigate calcium carbonate solutions at much lower concentrations than the solubility equilibria of the respective mineral phase is particularly appealing to study the influence of peptides on mineral deposition in a buffer system similar to biomineralizing compartments. It can be concluded that multichannel microfluidic SAW sensor systems are highly attractive for biomimetic mineralization studies in liquids and in real-time. It remains subject to further investigations whether this technique will bring the desired break-through in terms of elucidating the structure–function relationships in biomineralization. Our possiblities to replicate biominerals, to create sustainable processes and to design bio-inspired new biocompatible materials with sophisticated structures and functions will certainly expand.

## Experimental

### Biosensor measurements

A commercial biosensor system sam^®^X (SAW Instruments, Bonn, Germany) equipped with carboxyl-terminated self-assembled monolayer (SAM) coated Au sensor chips (SAW Instruments, Bonn, Germany) was used in this study. The surface acoustic wave phase signal as well as the amplitude signal was recorded for each experiment. Calibration procedures followed exactly as described according to the manufacturer's instructions. The instrument was operated at 22 ± 1 °C.

The software packages SensMaster und SequenceMaster (SAW Instruments, Bonn, Germany) were used to control the biosensor sam^®^X. Automated features were used for programming and documentation of the experimental conditions. The software package FitMaster (SAW Instruments, Bonn, Germany) was used to analyze the collected data and to electronically align, for example, the time of injection for comparative analyses of different experiments.

The phase signal is sensitive to both, mass changes on the surface as well as viscosity changes in the adjacent medium. Since the amplitude signal is influenced almost exclusively by the viscosity effect, a corrected phase signal can be calculated using the software SensMaster (SAW Instruments, Bonn, Germany). For this purpose, the phase signal is recorded after injection of 5% glycerin in the respective running buffer. The software extracts the corrected phase signal based on the assumption that glycerin induces a change in viscosity only, but does not affect the mass signal. All data as shown in this paper represent the original raw data, unless otherwise indicated.

### Statistics

OriginPro 8.6G (OriginLab Corporation, Additive GmbH, Friedrichsdorf, Germany) was used for data handling and statistics. Data from 5 measurements (120 measured values) in the time interval prior to the injection (−140 seconds to −100 seconds) were avaraged in order to account for deviations caused by the instrument and initial alignment procedures (e.g., shaded box in Figure 1C). For individual experiments, 120 values measured during injections, between 100–300 seconds, and after injections, between 700–900 seconds were averaged. The Wilcoxon signed-rank test and sign test were used to determine statistical significance (p-value 0.05).

### Solutions and buffers

Ultra-pure water with a conductivity of <0.026 µS (Barnstead™ GenPure™ Pro; Thermo Fisher Scientific) was used for all experiments including the preparation of solutions and buffers. The latter were prepared at room temperature (20 ± 3 °C).

#### Calcium carbonate solutions

Solid calcium carbonate (minimum 99.0%, Sigma-Aldrich, USA) was freshly dissolved at 100× the solubility product of 1.4 mg/L (20 °C) in pure water or in Gly–Gly buffers, which were previously adjusted to three different pH values pH 7.75, pH 8.2, or pH 9.0. The solid-free supernatant was used for experiments at 140 µmol/L and served as a stock solution for the respective dilution series.

#### Peptides in Gly–Gly buffer

A 10 mM stock solution of peptides AS8 and ES9 (99% purity; GeneCust, Dudelange, Luxembourg) [46] was prepared in Gly–Gly buffers (20 mM glycylglycine, 350 mM NaCl, 10 mM KCl), previously adjusted to either pH 7.75, 8.2 or 9.0. Each peptide was diluted in the respective buffers to final concentrations of 50 µM, 100 µM or 200 µM.

#### Citric acid

A 10 mM citric acid solution was prepared using 0.9% NaCl in pure water as the solvent. The pH was adjusted to pH 5.5 and diluted to a final concentration of 1 mM citric acid in order to reduce viscosity effects. This solution was also used in some experiments for regenerating the sensor chip after exposure to calcium carbonate.

#### Ethylendiamintetraacetate (EDTA) solution

For regenerating the sensor chip after exposure to calcium carbonate, 1 mM and 10 mM EDTA solutions were prepared from a 0.5 M EDTA stock solution, pH 8.0 (146 g/L).

#### Glycerol solution

For determining viscosity effects, a 5% glycerol solution was prepared in aqueous buffer.

## Supporting Information

File 1 Experimental details.

File 2 Phase signal calculation using glycerol as reference.

File 3 Monitoring cationic peptides ES9 and AS8 on COO-SAM, experimental values, Table S5.

File 4 Monitoring cationic peptides ES9 and AS8 on COO-SAM, experimental values, Table S6.

## References

[1] Lowenstam H A, Weiner S (1989). On Biomineralization.

[2] Weiner S, Addadi L (2011). Annu Rev Mater Res.

[3] Addadi L, Vidavsky N, Weiner S (2012). Z Kristallogr – Cryst Mater.

[4] Alberts B, Johnson A, Lewis J (2007). Molecular Biology of the Cell: Reference Edition.

[5] Mann S (2001). Biomineralization: Principles and Concepts in Bioinorganic Materials Chemistry.

[6] Knoll A H (2003). Rev Mineral Geochem.

[7] Raven J A, Knoll A H (2010). Geomicrobiol J.

[8] Gao H, Ji B, Jäger I L, Arzt E, Fratzl P (2003). Proc Natl Acad Sci U S A.

[9] Weiss I M, Marin F, Sigel A, Sigel H, Sigel R K O (2008). Met. Ions Life Sci. - Biomineralization: From Nature to Application.

[10] Meldrum F C, Cölfen H (2010). Nanoscale.

[11] Addadi L, Joester D, Nudelman F, Weiner S (2006). Chem – Eur J.

[12] Addadi L, Politi Y, Nudelman F, Weiner S, Novoa J J, Braga D, Addadi L (2008). Biomineralization design strategies and mechanisms of mineral formation: operating at the edge of instability. Engineering of Crystalline Materials Properties State of the Art in Modeling, Design and Applications.

[13] Falini G, Albeck S, Weiner S, Addadi L (1996). Science.

[14] Suzuki M, Iwashima A, Kimura M, Kogure T, Nagasawa H (2013). Mar Biotechnol.

[15] Marin F, Le Roy N, Marie B (2012). Front Biosci, Scholar Ed.

[16] Marie B, Joubert C, Tayalé A, Zanella-Cleón I, Belliard C, Piquemal D, Cochennec-Laureau N, Marin F, Gueguen Y, Montagnani C (2012). Proc Natl Acad Sci U S A.

[17] Marie B, Ramos-Silva P, Marin F, Marie A (2013). Proteomics.

[18] Levi-Kalisman Y, Falini G, Addadi L, Weiner S (2001). J Struct Biol.

[19] Miyamoto H, Endo H, Hashimoto N, Limura K, Isowa Y, Kinoshita S, Kotaki T, Masaoka T, Miki T, Nakayama S (2013). Zool Sci.

[20] Weiss I M (2012). Z Kristallogr – Cryst Mater.

[21] Meldrum F C, Cölfen H (2008). Chem Rev.

[22] Yang X, Xie B, Wang L, Qin Y, Henneman Z J, Nancollas G H (2011). CrystEngComm.

[23] Borah B M, Halter T J, Xie B, Henneman Z J, Siudzinski T R, Harris S, Elliott M, Nancollas G H (2014). J Colloid Interface Sci.

[24] Nielsen M H, Li D, Zhang H, Aloni S, Han T Y-J, Frandsen C, Seto J, Banfield J F, Cölfen H, De Yoreo J J (2014). Microsc Microanal.

[25] De Yoreo J J (2013). Research Methods in Biomineralization Science.

[26] Israelachvili J N (2011). Intermolecular and Surface Forces.

[27] Gebauer D, Kellermeier M, Gale J D, Bergström L, Cölfen H (2014). Chem Soc Rev.

[28] Verch A, Gebauer D, Antonietti M, Cölfen H (2011). Phys Chem Chem Phys.

[29] Perpeet M, Glass S, Gronewold T, Kiwitz A, Malavé A, Stoyanov I, Tewes M, Quandt E (2006). Anal Lett.

[30] Ruppel C C W, Fjeldly T A (2001). Advances in Surface Acoustic Wave Technology, Systems and Applications.

[31] Jung A, Gronewold T M A, Tewes M, Quandt E, Berlin P (2007). Sens Actuators, B.

[32] Hoummady M, Campitelli A, Wlodarski W (1997). Smart Mater Struct.

[33] Freund L B, Suresh S (2009). Thin Film Materials: Stress, Defect Formation and Surface Evolution.

[34] Andle J C, Vetelino J F (1994). Sens Actuators, A.

[35] Gronewold T M A (2007). Anal Chim Acta.

[36] Cui J, Iturri J, Götz U, Jimenez M, del Campo A (2013). Langmuir.

[37] Länge K, Rapp B E, Rapp M (2008). Anal Bioanal Chem.

[38] Joseph S, Gronewold T M A, Schlensog M D, Olbrich C, Quandt E, Famulok M, Schirner M (2005). Biosens Bioelectron.

[39] Kellermeier M, Cölfen H, Gebauer D (2013). Methods Enzymol.

[40] Weiss I M, Kaufmann S, Mann K, Fritz M (2000). Biochem Biophys Res Commun.

[41] Weber E, Guth C, Weiss I M (2012). PLoS One.

[42] Weber E, Bloch L, Guth C, Fitch A N, Weiss I M, Pokroy B (2014). Chem Mater.

[43] Gotliv B-A, Addadi L, Weiner S (2003). ChemBioChem.

[44] Pokroy B, Aizenberg J (2007). CrystEngComm.

[45] Pokroy B, Chernow V F, Aizenberg J (2009). Langmuir.

[46] Sengupta Ghatak A, Koch M, Guth C, Weiss I M (2013). Int J Mol Sci.

[47] Weiss I M, Schönitzer V, Eichner N, Sumper M (2006). FEBS Lett.

[48] Weiss I M, Lüke F, Eichner N, Guth C, Clausen-Schaumann H (2013). J Struct Biol.

[49] Tewes M, Glaß S, Schlensog M D, Quandt E (2005). Mass determination method for measuring the mass acting on a surface acoustic wave sensor, e.g., for use in micro-weighing scales, whereby a calibration is first carried out using an analyte. Ger. Pat. Appl..

