# Supplementary material for: Real-time monitoring of calcium carbonate and cationic peptide deposition on carboxylate-SAM using a microfluidic SAW biosensor
Source: Beilstein J Nanotechnol. 2014 Oct 22;5:1823–35. doi: 10.3762/bjnano.5.193 (PMC4222353; doi:10.3762/bjnano.5.193)
Supplement: File 1 — Experimental details. [file Beilstein_J_Nanotechnol-05-1823-s001.pdf]

Supporting Information File 1

for

Real-time monitoring of calcium carbonate and cationic peptide deposition on carboxylate-SAM using a microfluidic SAW biosensor

Anna Pohl[1,2] and Ingrid M. Weiss*[,1]

Address: [1]INM – Leibniz Institute for New Materials, Campus D2 2, 66123 Saarbrücken, Germany and [2]Saarland University, Campus D2 2, 66123 Saarbrücken, Germany

Email: Ingrid M. Weiss - ingrid.weiss@inm-gmbh.de

*Corresponding author

Experimental Details

Table S1: Sequence of injections on channel 1. Calcium carbonate interaction with COO-chip.

Order	Running buffer	Injection solution	Concen-tration	Volume	Flow rate	Channel				Time between injections
						1	2	3	4	
1.	H_2O	$CaCO_3$ in H_2O	17.5 µmol/L	400 µL	40 µL/min	X				5 minutes
2.	H_2O	$CaCO_3$ in H_2O	35 µmol/L	400 µL	40 µL/min	X				5 minutes
3.	H_2O	$CaCO_3$ in H_2O	70 µmol/L	400 µL	40 µL/min	X				5 minutes
4.	H_2O	$CaCO_3$ in H_2O	105 µmol/L	400 µL	40 µL/min	X				5 minutes
5.	H_2O	$CaCO_3$ in H_2O	140 µmol/L	400 µL	40 µL/min	X				5 minutes

Table S2: Calcium carbonate interaction with COO-SAM sensor chip at different flow rates. All injections were performed with 140 µmol/L calcium carbonate in pure water.

Order	Running buffer	Injection solution	Concen-tration	Volume	Flow rate	Channel				Time between injections
						1	2	3	4	
1.	H_2O	$CaCO_3$ in H_2O	140 µmol/L	400 µL	200 µL/min	X				5 minutes
2.	H_2O	$CaCO_3$ in H_2O	140 µmol/L	400 µL	100 µL/min	X				5 minutes
3.	H_2O	$CaCO_3$ in H_2O	140 µmol/L	400 µL	50 µL/min	X				5 minutes
4.	H_2O	$CaCO_3$ in H_2O	140 µmol/L	400 µL	25 µL/min	X				5 minutes
5.	H_2O	$CaCO_3$ in H_2O	140 µmol/L	400 µL	12.5 µL/min	X				5 minutes

Table S3: The running buffer was a 140 µmol/L solution of calcium carbonate in pure water. Different volumes of citric acid (1 mM) were injected as well as pure water. All experiments were performed on the same channel of the biosensor.

Order	Running buffer	Injection solution	Concen tration	Volume	Flow rate	Channel				Time between injections
						1	2	3	4	
1	$CaCO_3$ in H_2O	Citric acid	1 mM	100 µL	50 µL/min	X				5 minutes
2	$CaCO_3$ in H_2O	Citric acid	1 mM	200 µL	50 µL/min	X				5 minutes
3	$CaCO_3$ in H_2O	Citric acid	1 mM	400 µL	50 µL/min	X				5 minutes
4	$CaCO_3$ in H_2O	Pure water	-	100 µL	50 µL/min	X				5 minutes
5	$CaCO_3$ in H_2O	Pure water	-	200 µL	50 µL/min	X				5 minutes
6	$CaCO_3$ in H_2O	Pure water	-	400 µL	50 µL/min	X				5 minutes

Table S4: Experiment with peptides ES9 and AS8 in Gly-Gly buffer at different pH-values. Exactly the same order was maintained for all experiments performed in Gly-Gly pH 7.75 (channel 2), Gly-Gly pH 8.2 (channel 1) or, Gly-Gly 9.0 (channel 3). Peptide solutions with c(ES9) or c(AS8) = 200 µM in 20 mM Gly-Gly were used. c, concentration; V, injection volume; Δ*t*, time interval between injections, for which the microfluidic system was allowed to equilibrate using a Gly-Gly running buffer of the respective pH.

Order	Running buffer	Injection solution	c [mM]	V [µL]	Flow rate [µL/min]	Channel				Δ*t* [min]
						1	2	3	4	
1	Gly-Gly	EDTA	10	200	40	pH 8.2	pH 7.75	pH 9.0		5
2	Gly-Gly	Gly-Gly	20	200	40	pH 8.2	pH 7.75	pH 9.0		2
3	Gly-Gly	EDTA	10	200	40	pH 8.2	pH 7.75	pH 9.0		5
4	Gly-Gly	ES9	0.2	200	40	pH 8.2	pH 7.75	pH 9.0		2
5	Gly-Gly	EDTA	10	200	40	pH 8.2	pH 7.75	pH 9.0		5
6	Gly-Gly	AS8	0.2	200	40	pH 8.2	pH 7.75	pH 9.0		2
