# Supplementary material for: Real-time monitoring of calcium carbonate and cationic peptide deposition on carboxylate-SAM using a microfluidic SAW biosensor
Source: Beilstein J Nanotechnol. 2014 Oct 22;5:1823–35. doi: 10.3762/bjnano.5.193 (PMC4222353; doi:10.3762/bjnano.5.193)
Supplement: File 2 — Phase signal calculation using glycerol as reference. [file Beilstein_J_Nanotechnol-05-1823-s002.pdf]

Supporting Information File 2

for

Real-time monitoring of calcium carbonate and cationic peptide deposition on carboxylate-SAM using a microfluidic SAW biosensor

Anna Pohl[1,2] and Ingrid M. Weiss*[,1]

Address: [1]INM – Leibniz Institute for New Materials, Campus D2 2, 66123 Saarbrücken, Germany and [2]Saarland University, Campus D2 2, 66123 Saarbrücken, Germany

Email: Ingrid M. Weiss - ingrid.weiss@inm-gmbh.de

*Corresponding author

Phase signal calculation using glycerol as reference

Phase signal derived "normalized mass" calculation experiment by using glycerol injection for compensating solvent viscosity effects. A portion of 5% aqueous glycerol was injected after the equilibration of the system with the running buffer, in this case 140 µmol/L calcium carbonate. Supplementary Figure 1A chronologically shows four citric acid injections, followed by glycerol injection at tg = 4437 s. The phase and the amplitude signal represent the originally recorded data. The calculated overlay of the four citric acid injections (Suppl Fig. 1A, t1 = 2021 s; t2 = 2360 s; t3 = 2699 s; t4 = 3038 s) and the glycerol injection (Suppl Fig. 1A, tg = 4437 s), merged in Suppl. Fig. 1B-E at t = 0 is shown. Suppl. Figures 1B and 1C show the raw phase and amplitude signal of all injections whereby in Suppl. Figures 1D and 1E the calculated signals are presented.

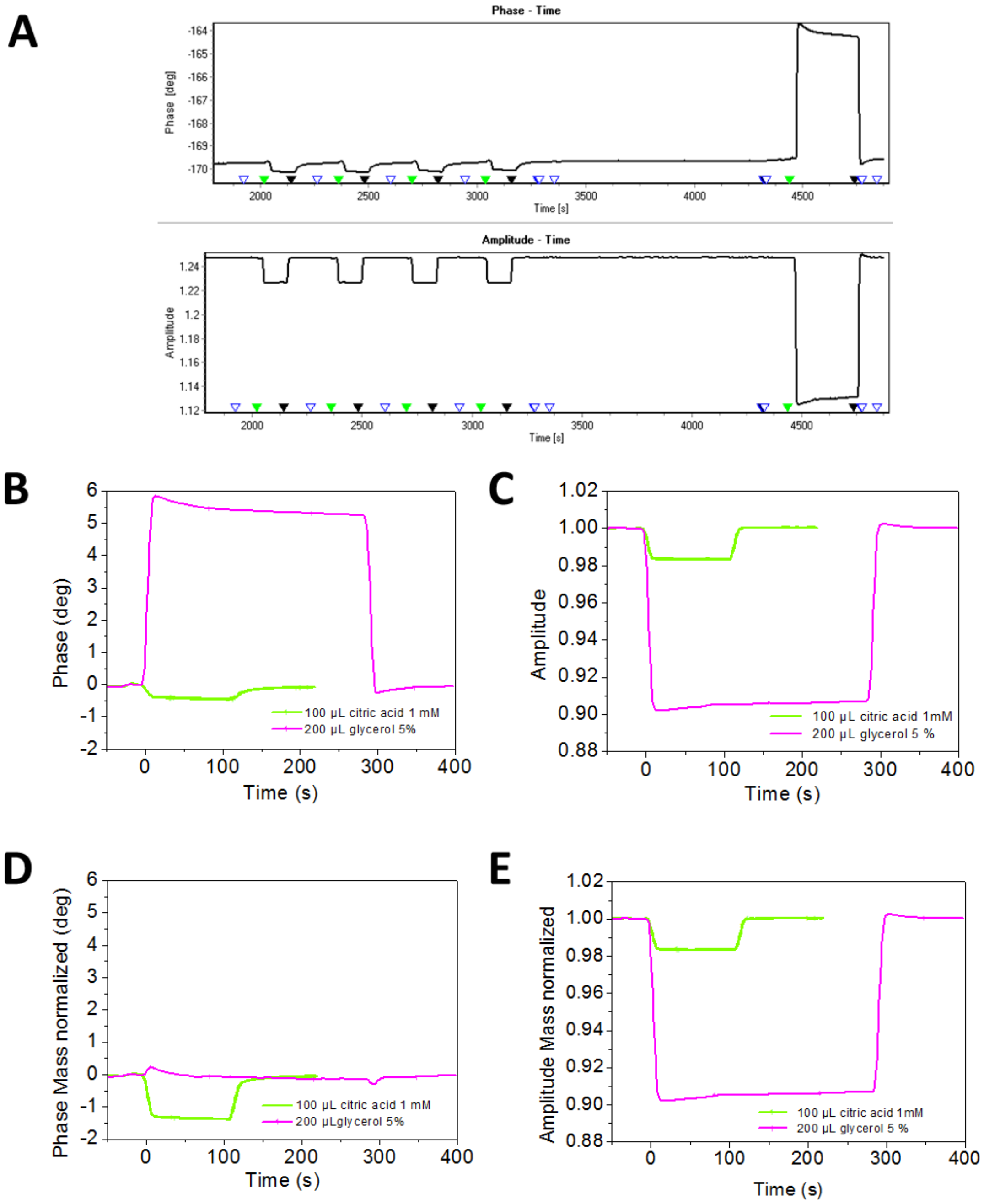

Figure S1: Experimental determination of the viscosity effect according to the established glycerol method previously described by the manufacturer. A, Phase and amplitude signal for 4 citric acid injections, followed by a 5% glycerol injection. Note that individual signals from the four citric acid injections cannot be distinguished in the overlay image. B-E, Comparison between the original signals (B,C) and calculated mass normalized phase signal (D) and corresponding amplitude signal (E). The latter is identical with the original amplitude signal (C).
