# Supplementary material for: Real-time monitoring of calcium carbonate and cationic peptide deposition on carboxylate-SAM using a microfluidic SAW biosensor
Source: Beilstein J Nanotechnol. 2014 Oct 22;5:1823–35. doi: 10.3762/bjnano.5.193 (PMC4222353; doi:10.3762/bjnano.5.193)
Supplement: File 3 — Monitoring cationic peptides ES9 and AS8 on COO-SAM, experimental values, Table S5. [file Beilstein_J_Nanotechnol-05-1823-s003.pdf]

Supporting Information File 3

for

Real-time monitoring of calcium carbonate and cationic peptide deposition on carboxylate-SAM using a microfluidic SAW biosensor

Anna Pohl[1,2] and Ingrid M. Weiss*[,1]

Address: [1]INM – Leibniz Institute for New Materials, Campus D2 2, 66123 Saarbrücken, Germany and [2]Saarland University, Campus D2 2, 66123 Saarbrücken, Germany

Email: Ingrid M. Weiss - ingrid.weiss@inm-gmbh.de

*Corresponding author

Monitoring cationic peptides ES9 and AS8 on COO-SAM, experimental values, Table S5

Comparative evaluation of the phase signal [°] for the two peptides. The phase signal was continously recorded for each measurement (3 replicates for each peptide at 3 pH values) and the difference between the phase value at the beginning of the peptide injection ($t < 0$ s, i.e. "-10 s") and after the peptide injection ($t = 310$ s) was calculated using the Origin 8.6 software package.

Table S5: Phase signal difference in [deg] (3 measurements, see e.g. Figure 6).

**Measurement**	**Peptide**	**pH 7.75**	**pH 8.2**	**pH 9.0**
1	AS8	0.14	0.05	0.05
2	AS8	0.30	0.41	0.19
3	AS8	0.24	0.24	0.06
Average	AS8	0.23 ± 0.082	0.23 ± 0.182	0.10 ± 0.077
1	ES9	0.09	0.04	0.05
2	ES9	0.12	0.11	0.08
3	ES9	0.09	0.08	0.03
Average	ES9	0.10 ± 0.018	0.08 ± 0.036	0.05 ± 0.027
