# Supplementary material for: Real-time monitoring of calcium carbonate and cationic peptide deposition on carboxylate-SAM using a microfluidic SAW biosensor
Source: Beilstein J Nanotechnol. 2014 Oct 22;5:1823–35. doi: 10.3762/bjnano.5.193 (PMC4222353; doi:10.3762/bjnano.5.193)
Supplement: File 4 — Monitoring cationic peptides ES9 and AS8 on COO-SAM, experimental values, Table S6. [file Beilstein_J_Nanotechnol-05-1823-s004.pdf]

Supporting Information File 4

for

Real-time monitoring of calcium carbonate and cationic peptide deposition on carboxylate-SAM using a microfluidic SAW biosensor

Anna Pohl[1,2] and Ingrid M. Weiss*[,1]

Address: [1]INM – Leibniz Institute for New Materials, Campus D2 2, 66123 Saarbrücken, Germany and [2]Saarland University, Campus D2 2, 66123 Saarbrücken, Germany

Email: Ingrid M. Weiss - ingrid.weiss@inm-gmbh.de

*Corresponding author

Monitoring cationic peptides ES9 and AS8 on COO-SAM, experimental values, Table S6

Comparative evaluation of the gradient of the phase signal [°] for the two peptides for the time of injections of the peptides ES9 and AS8. The phase signal was continously recorded for each measurement (3 replicates for each peptide at 3 pH values) and the slope of the linear fit of the phase value at the beginning of the peptide injection. Calculations were performed using the Origin 8.6 software package.

Table S6: Slope of the linear fit of the phase signal in [deg/s] (3 measurements, see e.g. Figure 6).

Measurement	Peptide	pH 7.75	pH 8.2	pH 9.0
1	AS8	0.053	0.106	0.072
2	AS8	0.044	0.074	0.047
3	AS8	0.041	0.075	0.056
Average	AS8	0.046 ± 0.006	0.085 ± 0.018	0.058 ± 0.013
1	ES9	0.01248	0.0723	0.0151
2	ES9	0.01308	0.01552	0.01348
3	ES9	0.01384	0.01439	0.01441
Average	ES9	0.01313 ± 0.00068	0.034 ± 0.033	0.01433 ± 0.00081
